# Enteric Fever and Related Contextual Factors in Bangladesh

**DOI:** 10.4269/ajtmh.18-0106

**Published:** 2018-07-25

**Authors:** Shampa Saha, Senjuti Saha, Rajib Chandra Das, A. S. G. Faruque, M. Abdus Salam, Maksuda Islam, Samir K. Saha

**Affiliations:** 1 Department of Microbiology, Child Health Research Foundation, Dhaka Shishu Hospital, Dhaka, Bangladesh;; 2 International Centre for Diarrhoeal Disease Research, Dhaka, Bangladesh;; 3 Bangladesh Institute of Child Health, Dhaka Shishu Hospital, Dhaka, Bangladesh

## Abstract

Enteric fever remains a major public health problem in the developing world. With the emergence of antimicrobial resistance, disease prevention is becoming essential. There is evidence that improvement of contextual factors, such as socioeconomic development and water supply and sanitation, reduce the burden of this disease. However, such positive results are not universal. This study describes enteric fever trends in Bangladesh along with these factors’ progress between 1990 and 2014. Retrospective enteric fever data were collected from Dhaka Shishu (children) Hospital (DSH), Shishu Shasthya Foundation Hospital (SSFH), International Center for Diarrheal Disease Research, Bangladesh, and Popular Diagnostic Center (PDC). Contextual factors data were gathered from relevant organizations and their websites and plotted against time to see trends. During 2001–2014, data for a total of 131,449 blood cultures were available at DSH, SSFH, and PDC. Of those, 7,100 (isolation rate 5.4%) yielded either *Salmonella enterica* serovar Typhi or *Salmonella enterica* serovar Paratyphi growth without visible change in isolation rate trends. Contextual factors data were reported from 1990 to 2014. There were significant developments for sanitation facilities, drinking water supply, female literacy, and reduction in poverty head count ratio. During this time period, population density also increased significantly. Despite improvements in these contextual factors in Bangladesh, the enteric fever trend seems steady, possibly because of high population density and unplanned development of water supply and sewerage system. Although proper development of these two factors is important, immunization with an effective vaccine is instrumental to prevent this disease immediately in endemic countries such as Bangladesh, specifically to overcome the challenge of emerging resistance to available antibiotics.

## INTRODUCTION

Enteric fever is a major public health problem in the developing world. Each year, enteric fever causes nearly 16 million illnesses and more than 153,000 deaths globally, most of which occurred in South Asia and sub-Saharan Africa.^1^ In South Asia, the incidence of enteric fever was 394.2 episodes per 100,000 person-years^2^ and in Bangladesh, the incidence of typhoid fever was reported to be 200 episodes per 100,000 person-years during 2003–2004.^3^ Two community-based studies that were conducted in the Kamalapur area of Dhaka during 2000–2001 and 2003–2004 reported that the blood culture positivity rates in febrile patients for enteric fever was 7.3%.^3,4^ A higher rate (9%) of blood culture positivity for enteric fever was reported by a tertiary hospital–based study during 2008–2013 in Dhaka.^5^ Another study at a private diagnostic center observed a similar rate (10%) of blood culture positivity for enteric fever during 1998–1999.^6^ A recent study from Bangladesh that investigated bacterial etiology of bloodstream infections reported *Salmonella enterica* serovar Typhi (*S.* Typhi) as the most frequently isolated organism, although they observed an overall decrease in the isolation rate of *S.* Typhi, especially the multidrug-resistant *S.* Typhi strains.^7^

This age-old disease, caused by *S.* Typhi and *Salmonella enterica* serovar Paratyphi (*S.* Paratyphi), was one of the largest killers during the pre-antibiotic era. Vaccines, although not perfect, against typhoid fever have been available since 1896.^8^ However, improvements in these vaccines were disrupted by the availability of antibiotics, which were remarkably successful in treating typhoid and saving lives; case fatality rates came down from 30% in the pre-antibiotic era to < 1% in the post-antibiotic era.^9^ Recently, the first conjugate vaccine to prevent typhoid fever has been prequalified by World Health Organization (WHO), and countries are now facing important decisions on the introduction of this vaccine.^10^ No vaccine has been developed to specifically target paratyphoid fever.

Meanwhile, multidrug-resistant (resistance to the first-line antimicrobials ampicillin, cotrimoxazole, and chloramphenicol) strains of *S.* Typhi and *S.* Paratyphi are on the rise, and antibiotics are no longer foolproof shots in treating enteric fever. Non-susceptibility to fluoroquinolones is almost omnipresent.^11^ Overall, the disease poses an imminent threat of widespread infections that may be as devastating as in the pre-antibiotic era.

Taking into consideration the fecal oral transmission of *S.* Typhi and *S.* Paratyphi, improvements in water, sanitation and hygiene (WASH) can aid in the prevention of contracting the *Salmonella* pathogens. In addition to WASH practices, socioeconomic status, environment, and consumption of contaminated foods have been considered as risk factors for contracting typhoid fever.^12^ Given that the typhoid vaccines have no protective effect on paratyphoid, a point receiving increasing attention is if the risk factors for typhoid are the same with respect to paratyphoid fever as incidence rates have increased in certain contexts where typhoid incidence has decreased.^12,13^ A study in Indonesia noted that *S.* Paratyphi bacteria must be present in higher concentrations than *S.* Typhi, suggesting contaminated food consumption could be a more important risk factor than unimproved water sources for paratyphoid.^14^ A review on enteric fever highlights that in-house factors such as hygiene practices, water source, and housing structure are key risk factors for typhoid fever, whereas paratyphoid fever risks are more strongly associated with external factors such as food purchasing habits (street vendors) and environmental risks (flooding).^12^ There are examples from Chile, Egypt, former Soviet Union, and Vietnam where typhoid and other enteric diseases have been controlled with the improvement of WASH and other contextual factors^15^; however, this is not always the case.^16^

In recent years, Bangladesh and other South Asian countries made good progress in all WASH-related and socioeconomic parameters. Bangladesh graduated as the low-and middle-income country (LMIC) with the fastest economic growth in the region. In this study, we aimed to describe the trend of enteric fever in conjunction with improvements in contextual factors over time in Bangladesh.

## METHODS

For this analysis, retrospective data on enteric fever and the contextual factors that may have had an influence on the occurrence of enteric fever were collected from different sources. Data for the last 25 years were requested from the related organizations/hospitals or searched on websites. All data included in this study were anonymized.

Ethics statement: Ethical approval was obtained from the ethical review committee of Bangladesh Institute of Child Health, Dhaka Shishu Hospital, Dhaka.

### Data on enteric fever.

Data on enteric fever were obtained from three hospitals and one diagnostic center located in Dhaka. The institutions under study were Dhaka Shishu (children) Hospital (DSH), Shishu Shasthya Foundation Hospital (SSFH), International Center for Diarrheal Disease Research, Bangladesh (icddr,b), and Popular Diagnostic Center (PDC). Dhaka Shishu (children) Hospital, established in 1972, is the largest pediatric hospital in the country. It has 640 beds and provides primary to tertiary care to children from all over the country. This government-aided private hospital treats 47% of patients free of cost and the rest with modest charges. Shishu Shasthya Foundation Hospital was established in 1983 and is the second largest pediatric hospital in Bangladesh with 200 beds. About 5% of the beds at SSFH are dedicated to those unable to pay for care. The laboratories of these two hospitals have been part of a laboratory network for GAVI’s Pneumococcal Vaccines Accelerated Development and Introduction Plan (2004–2008) and WHO-supported Invasive Bacterial Vaccine Preventable Diseases surveillance (2009–present) since 2004. International Center for Diarrheal Disease Research, Bangladesh initiated its activities in 1960 as a Cholera Research Laboratory and is currently one of the leading research institutes of Bangladesh, with an attached hospital. Popular Diagnostic Center is one of the largest outpatient-based private diagnostic centers in Bangladesh with a country-wide network. Three branches located in Dhaka city (Dhanmondi, English road, and Shantinagar branches) were included in this study.

At all sites, blood cultures were performed using standard methods^17,18^ and the blood culture method was constant over the reporting period. Two to three milliliters and three to five milliliters of blood was aseptically obtained from < 5 and > 5 year olds, respectively, for culture and inoculated into trypticase soy broth supplemented with sodium polyethanol sulphonate (0.25%) and isovitalex (1%). After incubation, blood culture bottles were subcultured on second, third, and fifth days of incubation. *Salmonella enterica* serovar Typhi/Paratyphi isolates were confirmed by standard biochemical tests and agglutination with *Salmonella* species and serovar specific antisera (RamelTM; Thermo Fisher Scientific, Inc., Waltham, MA).

### Data on contextual factors.

For the contextual factors, we considered any initiative that might have a direct or indirect influence on the occurrence of enteric fever. The included contextual factors are 1) access to improved water and sanitation, 2) poverty, 3) female literacy, and 4) population density. Access to improved sanitation facilities refers to the percentage of the population using such facilities that are likely to ensure hygienic separation of human excreta from human contact. They include flush/pour flush latrines (piped to sewer system, septic tank, and pit latrines), ventilated improved pit latrines, pit latrines with slab, and composting toilets.^19,20^ Similarly, access to an improved water source refers to the percentage of the population obtaining drinking water from sources that include piped water on premises (piped household water connection located inside the user’s dwelling, plot or yard), and other improved drinking water sources (public taps or standpipes, tube wells or boreholes, protected dug wells, protected springs, and rainwater collection).^19,20^ Adult female literacy rate was defined as the percentage of female population aged 15 years and older, who can both read and write, and understand a short simple statement about their everyday life.^21,22^ Poverty head count is defined based on the percentage of the population living below the global poverty lines ($1.90/day; 2011 international prices).^23^ Population density is the midyear population divided by land area in square kilometers. Population is based on the de facto definition of population, which counts all residents regardless of legal status or citizenship, except refugees.^24^ Data on contextual factors were obtained from different sources and are depicted in Table 1.

**Table 1:** Sources of data on contextual factors

Factor	Sources
Improved sanitation	WHO/UNICEF Joint Monitoring Program for Water Supply and Sanitation, World Bank^19,20^
Improved water supply	WHO/UNICEF Joint Monitoring Program for Water Supply and Sanitation, World Bank^19,20^
Poverty	Global Poverty Working Group, World Bank^23^
Female literacy	United Nations Educational, Scientific, and Cultural Organization (UNESCO) Institute for Statistics, World Bank^21,22^
Population density	World Bank, based on Food and Agriculture Organization and World Bank population estimates^24^

WHO = World Health Organization; UNICEF = United Nations International Children’s Emergency Fund.

As vaccines to prevent enteric fever are not available in the national Expanded Program on Immunization of Bangladesh, typhoid vaccination coverage was not considered as one of the contextual factors in this study.

### Statistical methods.

Rates of culture proved enteric fever cases were estimated for DSH, SSFH, and PDC using the number of blood culture–positive typhoid and paratyphoid cases as the numerator and the total number of blood cultures performed as the denominator. For icddr,b, the positivity rate of enteric fever was estimated using the number of stool culture–positive typhoid and paratyphoid cases as the numerator and the total admissions as the denominator. Enteric fever positivity rates and data on contextual factors were plotted by year to visualize longitudinal trends. Data were analyzed using STATA-13 (StataCorp. 2013. Stata Statistical Software: Release 13; StataCorp LP, College Station, TX).

## RESULTS

Data on enteric fever were available for different durations and from different sources (Table 2).

**Table 2:** Sources and characteristics of data on enteric fever

Source	Sample type	OPD/IPD	Age range	Reporting period
Dhaka Shishu (children) Hospital	Blood culture	OPD and IPD	0–16 years	2001–2014
Shishu Shasthya Foundation Hospital	Blood culture	IPD	0–17 years	2010–2014
Popular Diagnostic Center	Blood culture	OPD	0–82 years, 84% ≤ 16 years	2002–2014
International Center for Diarrheal Disease Research, Bangladesh	Stool culture	IPD	All ages	2001–2014

IPD = inpatient department; OPD = outpatient department.

**Figure 1.**
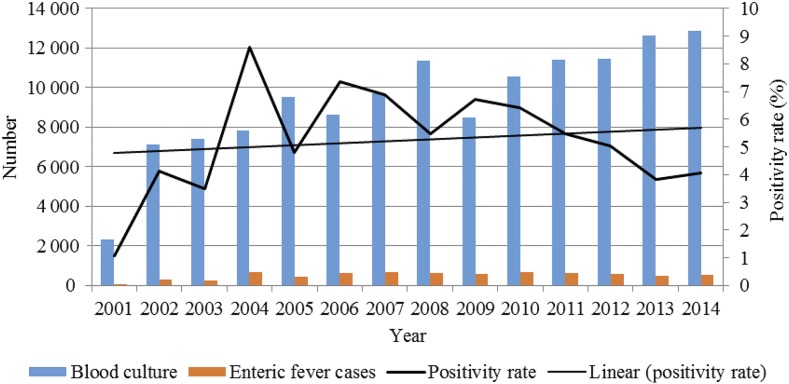
Number of blood cultures performed, enteric fever cases, and overall blood culture positivity rate of enteric fever. Blue bars show blood cultures performed across the study period. Orange bars represent the number of enteric fever cases detected across the study period. The dark black line shows the enteric fever positivity rate, which is the number of enteric fever cases out of blood cultures performed. The thin black line shows the linear trend of enteric fever positivity rate.

At DSH, SSFH, and PDC, data for a total of 131,449 blood cultures were available spanning 2001–2014. From those blood specimens, a total of 7,100 enteric fever cases were identified with an average isolation rate of 5.4% (Figure 1).

**Figure 2.**
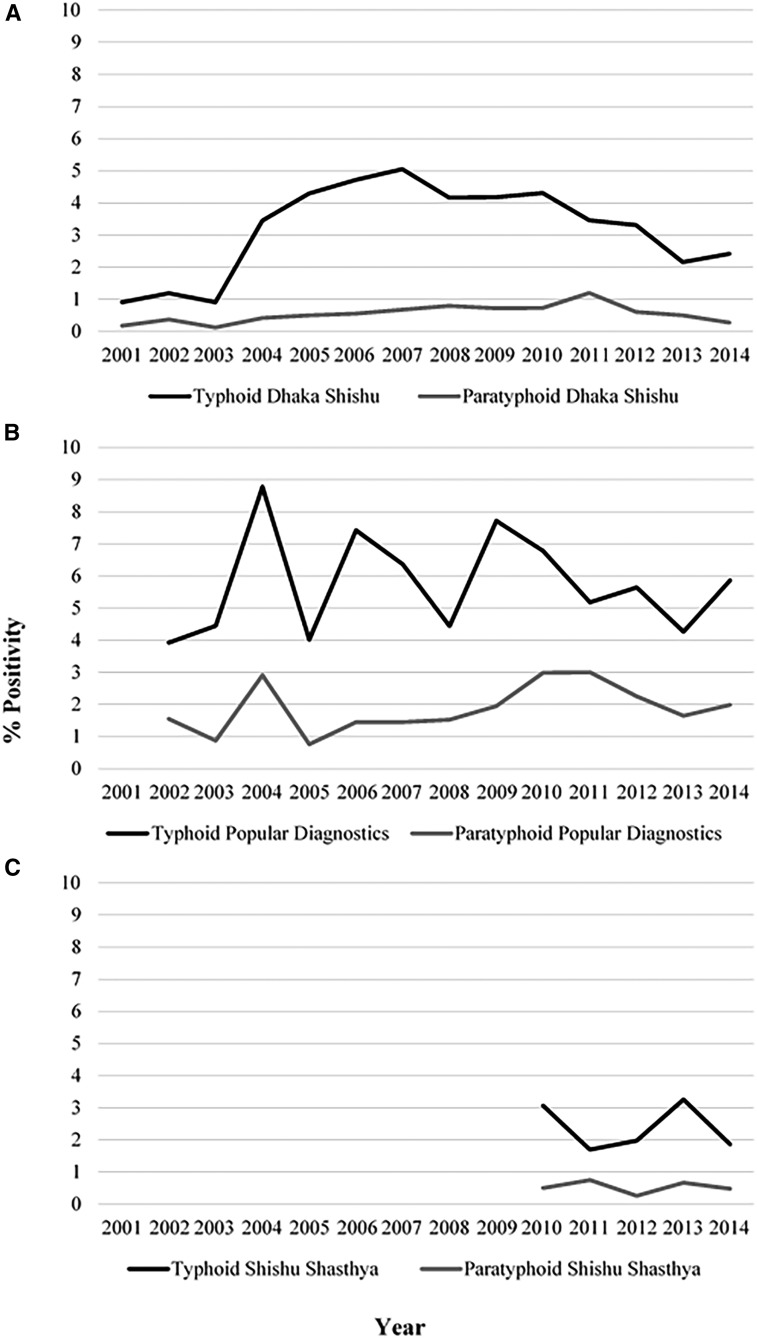
Positivity rate of enteric fever from blood cultures by year at Dhaka Shishu (children) Hospital, Shishu Shasthya Foundation Hospital, and Popular Diagnostic Center. Panel (**A**) shows the typhoid fever positivity rate trend at Dhaka Shishu Hospital as the black line. The gray line shows the paratyphoid positivity rate. Panel (**B**) shows the typhoid fever positivity rate trends in black from Popular Diagnostics and the gray line shows the paratyphoid fever positivity rate. Panel (**C**) shows the typhoid fever positivity rate trend as a black line for Shishu Shasthya Hospital. They paratyphoid positivity rate trend for Shishu Shasthya Hospital is shown in gray.

**Figure 3.**
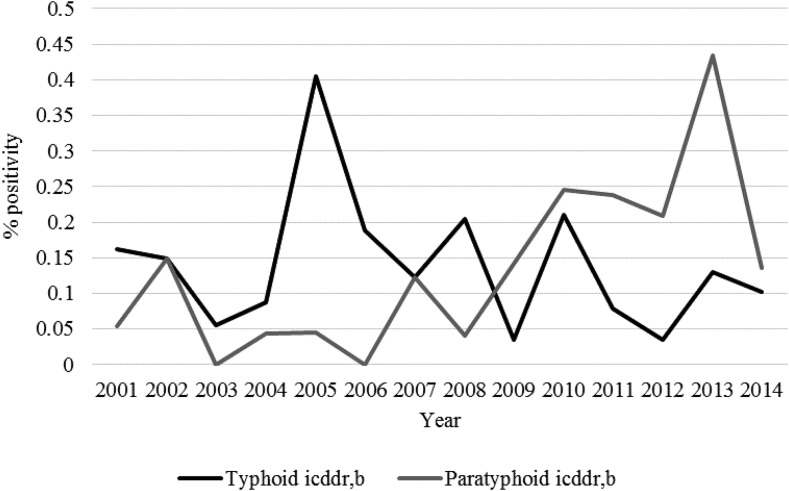
Positivity rate of enteric fever from stool cultures by year at International Center for Diarrheal Disease Research, Bangladesh (icddr,b). The black line shows the typhoid positivity rate trend at the icddr,b. The gray line shows the paratyphoid positivity rate trend at the icddr,b.

During 2001–2014, DSH identified 2,516 blood culture–positive enteric fever cases from both in and outpatient children. *Salmonella enterica* serovar Typhi isolation rates ranged from 0.9% in 2001 to its peak of 5.1% in 2007, whereas *S.* Paratyphi isolation rates ranged from 0.2% in 2001 to 1.2% in 2011 (Figure 2). The overall positivity rate of enteric fever was 3.9% (2,516/64,762; range: 1–5.7% by year). Shishu Shasthya Foundation Hospital identified 255 blood culture–positive enteric fever cases from the children who were hospitalized during 2010–2014. The positivity rate of *S.* Typhi ranged from 1.7% to 3.3% across the study period. In the same time period, *S.* Paratyphi positivity ranged from 0.3% to 0.75% in 2012 and 2011, respectively (Figure 2). The rate of enteric fever positivity was 2.9% per year (255/8,741). At PDC, the highest rates of *S.* Typhi and *S.* Paratyphi percent positivities were observed. Percent positivity rates ranged from 3.9% to 8.8% and 0.8–3% for *S.* Typhi and *S.* Paratyphi, respectively. Popular Diagnostic Center also reported the highest number of enteric fever cases (*N* = 4,329) from 2002 to 2014. The overall enteric fever positivity rate was highest at these centers; 7.5% per year (4,329/57,946) for overall enteric fever. In contrast to these institutions, icddr,b reported stool culture positive cases. A total of 93 stool culture positive cases were identified from 33, 534 hospitalized patients (0.28%). Percent positivity of *S.* Typhi by stool culture ranged from 0.04% to 0.4% in the period of 1993–2015. The positivity rate of *S.* Paratyphi ranged from 0% to 0.4% across the same period. Rates of culture-proved enteric fever cases per year at each hospital are shown in Figures 2 and 3.

**Figure 4.**
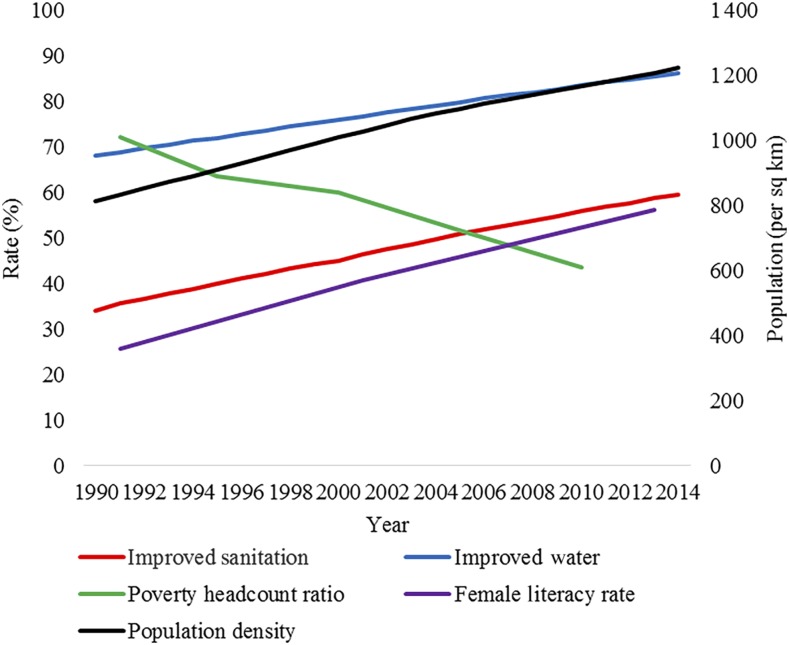
Longitudinal contextual factors in Bangladesh. The red line shows the percentage of the population in Bangladesh with access to improved sanitation facilities. The blue line shows the percentage of the population in Bangladesh with access to improved water sources. The green line shows the poverty head count ratio, which is the percentage of the population living on less than $1.90/day. The purple line shows the percentage of adult female literacy (15 years and older) in Bangladesh. The black line uses the secondary axis on the graph and shows population density per square kilometer.

For this report, data for contextual factors were available from 1990 to 2014, but enteric fever data were only available from 2001 to 2014. We selected a longer duration for the contextual factor data to visualize the trend in the selected factors. From 1990 to 2014, upward trends have been observed for improved sanitation facilities, improved drinking water sources, adult female literacy, and population density (Figure 4). The percentage of the population using improved sanitation facilities increased from 34% to 59.6% during this period. Similarly, the percentage of the population using improved drinking water sources rose from 68% to 86.2%. Data on female literacy rate were available for 1991, 2001, and 2013 only. From 1991 to 2013, the literacy rate of women aged 15 years or more increased from 25.8% to 56.2%. On the other hand, a decreasing trend was observed for poverty head count ratio, which decreased from 72.2% to 43.7% during 1991–2010. Within the same time period, the population density in Bangladesh increased from 814 to 1,222 people per square kilometer.

## DISCUSSION

The study showed that all the components of WASH and other socioeconomic parameters of Bangladesh have improved significantly. These improvements in sanitation, drinking water, female literacy, and reduction in poverty are expected to have a direct impact on reducing the transmission of enteric fever and other communicable diseases.^14,25^ However, enteric fever was a major cause of bloodstream infection, and the blood culture positivity rate for enteric fever seems to be unchanged.

This study showed a lower isolation rate (5.4%) for enteric fever from blood over the period 2001–2014 compared with the studies conducted earlier. This lower rate of positivity may be due to increasingly high use of antibiotics before seeking care, specifically with wide availability of oral antibiotics (azithromycin and cefixime) throughout the country. Another possible reason could be advising more blood culture for quick screening at the study sites, with the increased availability of results within shortest turnaround time.^17^

Overall, there are no visible changes in the trend of enteric fever positivity rate in Bangladesh, despite the apparent improvement in water, sanitation, hygiene, and other socioeconomic parameters. A possible reason of erosion of expected benefit from improving WASH factors is the high population density, which has increased from 814 to 1,222 people per km^2^, along with other contextual growths.^24^ In densely populated areas such as Dhaka, available resources are limited and might make people living in these areas vulnerable to ill-health.^26^ Two randomized control trials in India showed minimal impact of improved latrine coverage on diarrhea rates in children < 5 years of age and fecal contamination of water stored in the household.^16,27^ This was also seen in a recent study in Bangladesh, which showed that interventions to improve water quality and personal hygiene yielded limited success in preventing severe cholera, another enteric disease.^28^ This is possibly due to the rapid urbanization resulting in the high population density in urban areas.^19^ Although the infrastructure for water supply, availability of toilets, waste disposal system, etc., have been introduced, most of these developments were performed very rapidly and prevention of waterborne diseases was not taken into consideration. Furthermore, proper sanitation, specifically sewerage system and water supply, bears a huge price tag^29,30^ and thus most of the low- and middle-income countries (LMICs) implement their systems with limited resources. The resource limitation along with fast population growth leads to building too much and too fast making the “ship vulnerable to sink.” All in all, unplanned and improper WASH system facilitates the dissemination of enteric pathogens in densely populated urban populations. In a recent study, we have seen that 66% of tap water samples of study sites in Dhaka are positive for *S.* Typhi/Paratyphi DNA (*N* = 39), as detected by quantitative polymerase chain reaction.^31^ In addition, we also observed complex dynamics in antimicrobial susceptibility patterns of typhoid, including high rates of non-susceptibility to fluoroquinolone (data not shown).

Our study has several limitations. Different levels of data on enteric fever were reported by the participating hospitals for different age groups and durations. The data generated from the studies were not harmonized, so the inclusion and exclusion criteria were different, and blood collection was performed mostly at the discretion of the respective physicians. The numerator and denominator were different for icddr,b, which did not allow us to combine all the data together for analysis. However, collection of multiyear data from different sources, multiple hospitals, inpatient departments, outpatient departments, private diagnostic centers, and research institutes significantly strengthens the study findings of high enteric fever burden.

Our data showed a steady trend of enteric fever positivity rate in Bangladesh. The blood culture positivity rate can be used as a proxy for enteric fever disease burden if the method for case selection for blood culture and detection of *S.* Typhi/Paratyphi at laboratory remain stable. This is the best we can do in resource-poor endemic countries—analyzing routine blood cultures. Establishing specific enteric fever surveillance is resource intensive, expensive, and hard to sustain. Having said that, there are no new studies in place like Surveillance for Enteric Fever in Asia Project and Severe Typhoid Fever Surveillance in Africa—which in some countries are looking specifically at enteric fever. In future, comparison with data derived from those studies can give us more insight on suitability of blood culture positivity rate as a proxy of typhoid disease burden.

## CONCLUSION

In conclusion, enteric fever remains a serious public health concern in Bangladesh in spite of significant development in the country. Implementation of WASH is always expensive, and it is more complex and time consuming for a city where water supply and sewerage systems are already in place. The implementation of this initiative is also challenging because of the involvement of multiple stakeholders. Any work on WASH components in Bangladesh involves multiple ministries and organizations. Therefore, it will be a challenge to overhaul the whole system of water supply and sewerage system to improve the scenario. Moreover, the improvements in contextual factors with enteric fever remaining endemic suggest that improvements in these factors alone are not sufficient to eradicate typhoid and paratyphoid fever. An appropriate vaccine, specifically a conjugate vaccine that is effective in young children, can prevent this disease within a short period of time. It is specifically true for Bangladesh as the country is GAVI eligible and has a very high-performing immunization program with 91% coverage of pentavalent vaccine in place.^32^ With imminent decisions to be made about introductions of the recently prequalified typhoid conjugate vaccine, Bangladesh is generating data on burden of enteric fever, as the pre-vaccine baseline data, to determine the impact of the vaccine in the post-vaccine era.
